# Acoustic schwannomas are often accompanied with disproportionately enlarged subarachnoid space hydrocephalus.

**DOI:** 10.1186/2045-8118-12-S1-O33

**Published:** 2015-09-18

**Authors:** Hisayuki Murai, Toshimasa Shin, Yoshinori Higuchi, Naokatu Saeki

**Affiliations:** 1 Chiba Univeristy Graduate School of Medicine, Japan

## Introduction

Hydrocephalus with tight high convexity and wide Sylvian fissure is called disproportionately enlarged subarachnoid space hydrocephalus (DESH). DESH on MRI is thought to be one of the characteristic features of idiopathic normal pressure hydrocephalus (iNPH) and useful to differentiate from other ventriculomegalic status. On the other hand schwannomas are often complicated by communicating hydrocephalus, among which the expansion of the Sylvian fissure is often observed. So we examined relationship between acoustic schwannoma and communicating hydrocephalus or DESH.

## Methods

In 178 acoustic schwannomas those are followed in our institute between April 2000 and January 2015, 19 patients, were excluded for insufficient image studies. A total of 159 cases were devided into two groups. A group without 4th ventricle deformation, its tumor size is approximately less than 3cm, comprises 122 cases. The other group with 4th ventricle deformation, its tumor size is approximately larger than 3cm, was 37 cases. In each group, the incidence of hydrocephalus or DEH was studied and was compared with the findings of iNPH epidemiological studies. Hydrocephalus was classified into DESH like and non-DESH like on CT or MRI by 2 observers. We also studied the context of a spinal fluid protein in 10 cases and spinal CSF outflow resistance in 4 cases.

## Results

Hydrocephalus was observed in 37 (23%) out of 159 total cases. Number of DESH like hydrocephalus was 12 and non-DESH like case was 20. In 5 cases only CT scan was made and they were excluded from further analysis. In the group with 4th ventricle deformation, 18 cases (49%) presented hydrocephalus. And 5 cases (28%) showed DESH like features. On the other hand, in the group without 4th ventricle deformation the occurrence of hydrocephalus was 11% (14/122 cases). And DESH like features were observed in 50% of cases (7/14 cases). Measurement of spinal fluid proteins were conducted in 10 cases, mean value was 87 mg/dl in DESH group (6 cases) and 44 mg/dl in a non-DESH group (4 cases). Protein content was higher in DESH group, but it was not significant (p=0.075). In four cases with DESH features spinal CSF outflow resistance was measured and elevated in all cases.

## Conclusions

Acoustic schwannomas are often complicated with hydrocephalus even in small tumor size. The incidence of hydrocephalus in group without 4th ventricle deformation is significantly higher than the general population. And DESH like findings are more common in hydrocephalus associated with small tumors. As a mechanism to exhibit DESH like features, the possibility of solute load on the CSF has been suggested, but it requires further consideration.

